# Protein phosphatases regulate growth, development, cellulases and secondary metabolism in *Trichoderma reesei*

**DOI:** 10.1038/s41598-019-47421-z

**Published:** 2019-07-29

**Authors:** Aroa Rodriguez-Iglesias, Monika Schmoll

**Affiliations:** Austrian Institute of Technology GmbH, Health & Environment, Bioresources, Konrad-Lorenz-Straße 24, 3430 Tulln, Austria

**Keywords:** Fungal genetics, Molecular biology

## Abstract

*Trichoderma reesei* represents one of the most prolific producers of plant cell wall degrading enzymes. Recent research showed broad regulation by phosphorylation in *T. reesei*, including important transcription factors involved in cellulase regulation. To evaluate factors crucial for changes in these phosphorylation events, we studied non-essential protein phosphatases (PPs) of *T. reesei*. Viable deletion strains were tested for growth on different carbon sources, osmotic and oxidative stress response, asexual and sexual development, cellulase and protease production as well as secondary metabolism. Six PPs were found to be positive or negative regulators for cellulase production. A correlation of the effects of PPs on protease activities and cellulase activities was not detected. Hierarchical clustering of regulation patterns and phenotypes of deletion indicated functional specialization within PP classes and common as well as variable effects. Our results confirmed the central role of catalytic and regulatory subunits of PP2A which regulates several aspects of cell growth and metabolism. Moreover we show that the additional homologue of PPH5 in *Trichoderma* spp., PPH5-2 assumes distinct functions in metabolism, development and stress response, different from PPH5. The influence of PPs on both cellulase gene expression and secondary metabolite production support an interrelationship in the underlying regulation mechanisms.

## Introduction

Adaptation to environmental conditions for survival and successful competition in nature requires an efficient machinery to detect signals as availability of nutrients, light or the presence of a potential mating partner or competitor. Perception, transmission and subsequent cellular responses are regulated by post-translational modifications such as phosphorylation and dephosphorylation^1^.

*Trichoderma reesei* is the major producer of cell wall degrading enzymes, an industrial source for the use of renewable lignocellulosic material towards production of biofuels and biorefineries^2^. Nevertheless, investigation of the impact of signal transduction pathways on biosynthesis of cell wall degrading enzymes has not been done in detail and it is still in its beginnings^3^.

Mechanisms involved in regulation of expression of cell wall degrading enzymes in *T. reesei* have been studied in detail over decades, in concern to carbon-source-dependent regulation pathways^4,5^. Posttranslational modifications like phosphorylation often regulate protein function, protein turnover, protein-protein interactions as well as intracellular signal transduction^6,7^. Concerning transcription factors, phosphorylation of CRE1 (carbon catabolite repressor 1) and ACE2 (activator of cellulases 2) are required for binding to their target sequences^8,9^. Moreover, analysis of the amino acid sequences of several transcription factors (XYR1 (xylanase regulator 1), ACE1 (activator of cellulases 1), ACE2, HAP2/3/5 (HAT associated proteins) and CRE1) have led to identification of phosphorylation sites for cAMP dependent protein kinase, casein kinase II and protein kinase C^10^. Putative XYR1 phosphorylation sites have been also identified *in silico*. Recently, a broad phosphoproteomic study^11^ additionally confirmed phosphorylation of the regulators HAP2^12^, ACE1^13^, VEL1 (Velvet 1)^14^, VIB1 (vegetative incompatibility blocked)^15,16^, XPP1 (xylanase promotor binding protein 1)^17^. Accordingly, several of the mutations found in cellulase hypersecreting fungi created by random mutagenesis target genes involved in post-transcriptional processes^18–20^. However, neither the kinases nor the protein phosphatases counteracting the activity of these kinases have been identified so far. The relevance of phosphorylation in carbon source signaling is already known in filamentous fungi^11,21^ and yeast^22–25^. For example, the transcription factor XlnR (xylanase regulator) in *Aspergillus oryzae*, which is a transcriptional activator in the induction of xylanolytic and cellulolytic enzymes, is regulated by reversible phosphorylation^26^. Cellulase and hemicellulase production in *A. nidulans* is modulated by seven PPs, resulting in reduced endocellulase activity when deleted^27^. Consequently, protein phosphatases crucially impact carbon-source signaling, metabolic processes and enzyme expression in fungi, which warrants detailed investigation.

Light represents an important environmental cue, which has a broad influence on metabolic pathways in fungi^28^ and considerably influences regulation of plant cell wall degrading enzymes^29^. The circadian clock of fungi coordinates a daily rhythmicity of gene expression and phosphorylation tunes half-life, subcellular localization, transcriptional activity and conformation of clock components^30,31^. The Frequency (FRQ) protein is negative regulator of the heterodimeric photoreceptor complex formed by white collar-1 and white collar-2 in *N. crassa*. In *T. reesei* and in *N. crassa* the transcription factors forming this White Collar Complex (WCC) or the *T. reesei* equivalent, the Blue light regulator complex (BLRC), regulate cellulase gene expression^32–34^. FRQ is sequentially phosphorylated in the course of a circadian day in order to measure time and establish a circadian rhythm^35,36^ and hence represents an interesting example for phosphorylation dependent regulation. Thereby, kinases and phosphatases act in balancing the phosphorylation status of FRQ and WCC. The actions of CK-1a (casein kinase 1a) and CKII (casein kinase II) over the WCC phosphorylation status are counteracted by PP2A and PP4 phosphatases in *N. crassa*. The half-life of FRQ increases as a result of dephosphorylation by PP1 and PP4. Interestingly, although PP2A can be deactivated by phosphorylation, it harbors auto-dephosphorylation activity and it can rapidly reactivate itself^37^. PP2A, besides its role in the maintenance of circadian rhythmicity, is also involved in rapamycin (TOR; target of rapamycin) signaling and may also be regulatorily connected to cAMP-PKA signaling^25,38^. PP1 phosphatases also play a role in a broad range of cellular functions, including glycogen metabolism, cell-cycle progression^39–41^.

Response to white light was shown to be linked to oxidative stress response in *T. reesei* via Cys96 of the photoreceptor ENV1. A mutation of this cysteine ENV1 interferes with the stress response in light^42^. Also in *T. atroviride*, crosstalk of carbon metabolism, light response and oxidative stress response was shown^43,44^.

Generally speaking, phosphorylation acts as the currency of signaling in transduction pathways. The significance of phosphorylation is reflected by the fact that kinases and phosphatases genes of *S. cerevisiae*, which represent only 6% of the genome of yeast, modify 30% of the proteome^45^. For genes encoding the responsible enzymes for (de)phosphorylation, protein kinases and protein phosphatases (PPs) in *T. reesei* a detailed manual annotation is available^46^. Briefly, PPs are classified based on substrate specificity, sequence homology and structural characteristics. This classification results in two major groups, serine/threonine protein phosphatases (PSP) and tyrosine protein phosphatases (PTP)^47^. The PSPs are classified into the protein phosphatase P (PPP) family, which includes multimeric proteins as PP1 (PPZ), PP2A, PP2B and PP5 groups, and the protein phosphatase M (PPM) family, which includes the PP2C, and the third group of aspartate-based phosphatases. The PTP group comprises the specific tyrosine protein phosphatases (PTPs), dual specificity protein phosphatases (DSPs), low-molecular-weight protein tyrosine phosphatases (LMW-PTPs) and the *cdc25* phosphatases (CDC25). The group of low-M_r_ protein Tyr phosphatases (LMW-PTPs) belongs to an evolutionarily distinct category, whose members have converged on a similar catalytic mechanism^48,49^. Previously it was assumed that there are no tyrosine kinases in fungi^50^. Nevertheless, recently tyrosine residues were found to be phosphorylated in proteins from *T. reesei*^11^, indicating that besides the annotated protein tyrosine phosphatases also kinases phosphorylating tyrosine residues should be present in fungi. Besides catalytic subunits, there are protein phosphatase regulatory subunits that tune protein phosphatase specificity and function^51^. Furthermore, there are proteins regulating the activity of protein phosphatases, like phosphotyrosyl phosphatase activators (PTPA, also known as PP2A phosphatase activator) and phosphatase inhibitors.

After annotation of genes encoding PPs in the genome of *T. reesei*^46^, we obtained 15 viable phosphatase mutants and studied their phenotype in order to elucidate the function of the corresponding proteins. Phenotype assays revealed functions of PPs in growth, development, osmotic and oxidative stress response and secondary metabolite production. In many cases the effects caused by the deletions occurred in a light dependent manner, showing that protein phosphatases in part have a different relevance for fungal physiology in light than in darkness. Moreover, for some PPs functions in regulation of protease and cellulase production was observed. Additionally, we provide the first characterization of an additional homologue of PPH5 in *T. reesei*, which we designated PPH5-2.

## Results and Discussion

### Regulation patterns of phosphatase genes under different conditions

To gain an overview on regulation of phosphatase genes at a transcriptional level, we analyzed the transcription patterns of protein phosphatase encoding genes in available transcriptome data. Genes specifically regulated under a certain condition are expected to play a regulatory role under this condition. Additionally, regulation of a certain gene in a mutant strain indicates an importance of this gene for the phenotype and the regulatory alterations in this mutant. Moreover, co-regulated genes are likely to have functions in the same pathway under the given conditions. This correlation can also be explained by the double-lock mechanism in which a major regulator impacts transcription factors as well as their target genes^52^, as for example with regulation of *xyr1* by CRE1^53,54^.

We analyzed protein phosphatase transcript patterns in photoreceptor mutants^55^, in mutants of the heterotrimeric G-protein pathway^56^ reflecting nutrient sensing and upon growth on different inducing (cellulose, lactose, sophorose) and repressing/non inducing (glucose, glycerol) carbon sources [56]. The effects of components of the G-protein pathway on cellulase gene expression have already been studied in *T. reesei* and *N. crassa*^56–59^. Besides sensing of pheromones^60–62^, also different nutrients are sensed by fungi via G-protein coupled receptors (GPCRs)^63^. For *T. reesei* of those nutrients only glucose was shown so far to be sensed by GPCRs^64^. Additionally, we compared expression patterns under conditions of sexual development and growth on cellulose^65,66^.

**Figure 1:**
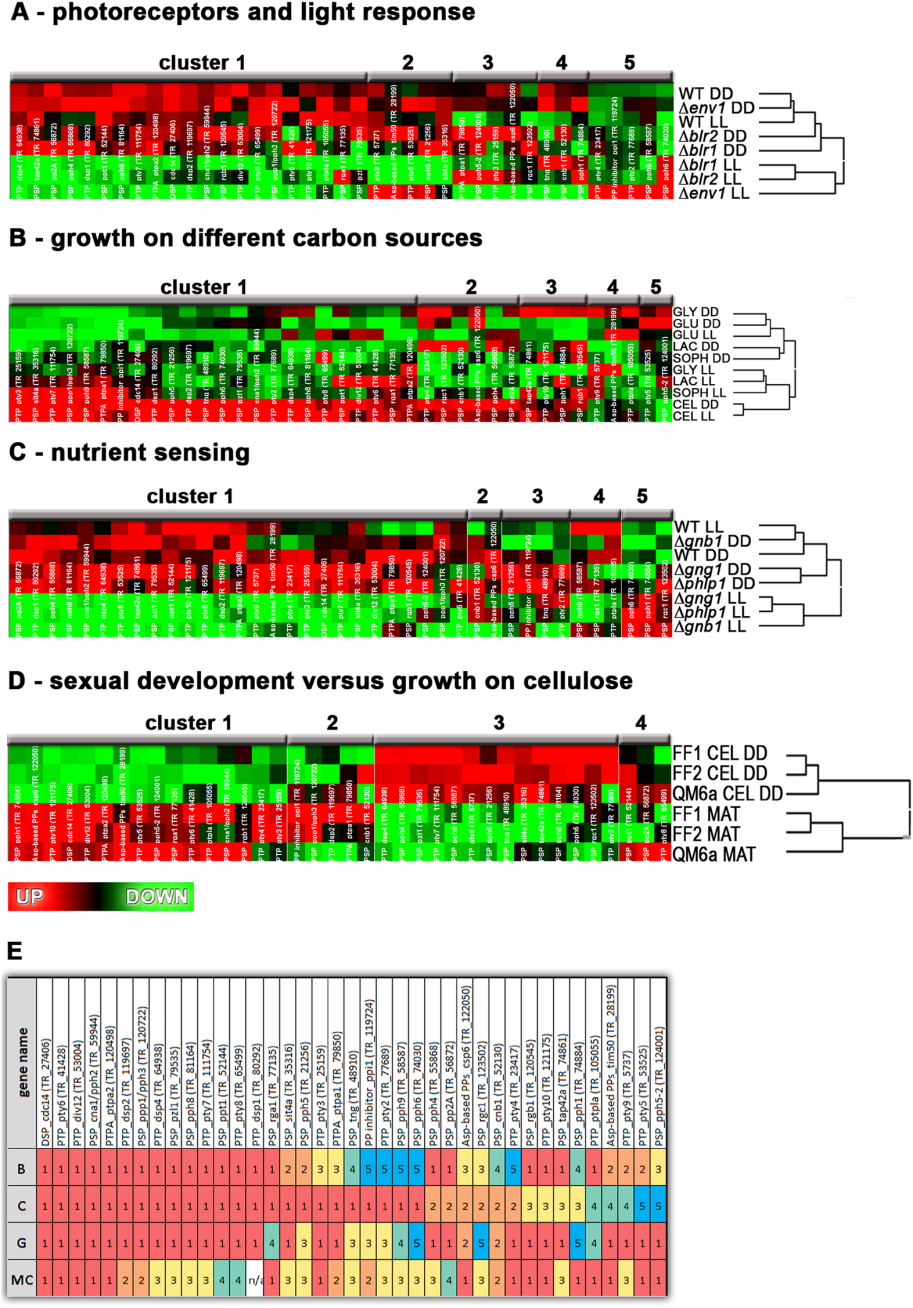
Analysis of transcription patterns of annotated protein phosphatase genes. Available transcriptome data from different conditions were used for hierarchical clustering. (**A**) Transcript patterns of PP genes in wildtype in constant light (LL) and constant darkness (DD) as well as in photoreceptor mutants upon growth on Mandels Andreotti medium with cellulose as carbon source^55^. (**B**) Transcript patterns of PP genes in wildtype grown on inducing (cellulose (CEL), lactose (LAC), sophorose (SOPH)) and non-inducing/repressing carbon sources (glycerol (GLY), glucose (GLU)) in constant light and constant darkness^64^. (**C**) Transcript patterns of PP genes in strains lacking components of the heterotrimeric G-protein pathway implicated in nutrient sensing^56^. (**D**) Transcript patterns of PP genes in female fertile strains and the female sterile QM6a upon growth on cellulose (CEL) in darkness and under conditions of sexual development (MAT) in daylight^66^. (**E**) Assignment of genes to the same cluster as shown by similar colors and numbers indicates co-regulation under the respective condition. For TR_80202 no data are available on transcript levels in the dataset of cellulose compared to mating (n/a). Abbreviations: B: blue light regulation, C: carbon regulation, G: regulation by the heterotrimeric G-protein pathway, MC: Regulation by conditions of cellulose production or mating. A high resolution figure is provided as Additional file 3.

These analyses showed that depending on the conditions, transcript patterns or individual protein phosphatase genes changed forming clusters specific for the investigated condition (Fig. 1A–D), although quite some overlap in assignment to cluster 1 is obvious (Fig. 1E). The different clusters contained protein phosphatase genes of different groups, indicating functional specialization within every group. Co-regulation of different protein phosphatases suggests that groups of protein phosphatases act on their target pathways in different combinations depending on the environmental conditions as reflected by coregulation in signaling mutants (Fig. 1).

In particular, several genes showed statistically significant regulation by ENV1 (envoy 1), including a gene encoding a phosphatase inhibitor (*ppi1*/TR_119724; 4.5fold up-regulated in ∆*env1*). The PTP encoding gene *pty2*/TR_77689 and a gene encoding a LMW-PTP (*pty4*/TR_23417) are up-regulated in ∆*env1* as well (>2fold) and four PP genes are down regulated in this strain between 2 and 3fold (*pty10*/TR_121175, *dsp2*/TR_119697, *cdc14*/TR_27406 and *pty8*/TR_65499). Thereby, *pty8* is also downregulated in strains lacking *blr1* or *blr2* in light and TR_79635 is down-regulated in ∆*blr1* in light^55^.

Regulation by subunits of the heterotrimeric G-protein pathway occurs for *pty8*/TR_65499 in light in strains lacking *phlp1*, *gnb1* or *gng1*. Additionally, the *pty9*/TR_5737 gene encoding a PTP shows decreased transcript levels in light in ∆*phlp1* and *ptpa2*/TR_120498 under the same conditions in ∆*gnb1*^56^.

Interestingly, no significant regulation of these genes has been detected between growth in light and in darkness in available transcriptome data. Regulation of protein phosphatase encoding genes by either photoreceptors or components of the heterotrimeric G-protein pathway only occurs in light. Consequently, a light dependent relevance of PPs for the physiology of *T. reesei*, that is mediated by photoreceptors and the G-protein pathway is likely. PP genes were not among the genes regulated in an induction specific manner^64^, indicating that their function in metabolic processes or cabon utilization depends on posttranslational regulation including (de) activation. Accordingly, phosphorylation of *dsp2*/TR_119697, *rga1*/TR_77135 and *sit4a*/TR_35316 was confirmed for *T. reesei*^11^.

### Generation of knock-out mutants of protein phosphatases

Our efforts to delete the genes annotated as protein phosphatases and related genes^46^ resulted in viable knock out strains for 12 catalytic PP subunits and 3 regulatory PP subunits. In many cases when no viable mutants could be recovered, their homologs in *N. crassa* were essential as well, since the corresponding transformants in *N. crassa* could only be recovered as heterokaryons (Additional file 1; Supplementary Table S1). The respective genes are consequently assumed to be essential in *T. reesei* as well. For PP genes that are not essential in *N. crassa* transformation for gene deletion was repeated at least three times and it did not result in viable mutants in *T. reesei*.

The obtained strains were analyzed for phenotypes under diverse conditions in order to elucidate their functions and identify common pathways on which more than one PP acts.

### Protein phosphatases are important for normal growth

**Figure 2:**
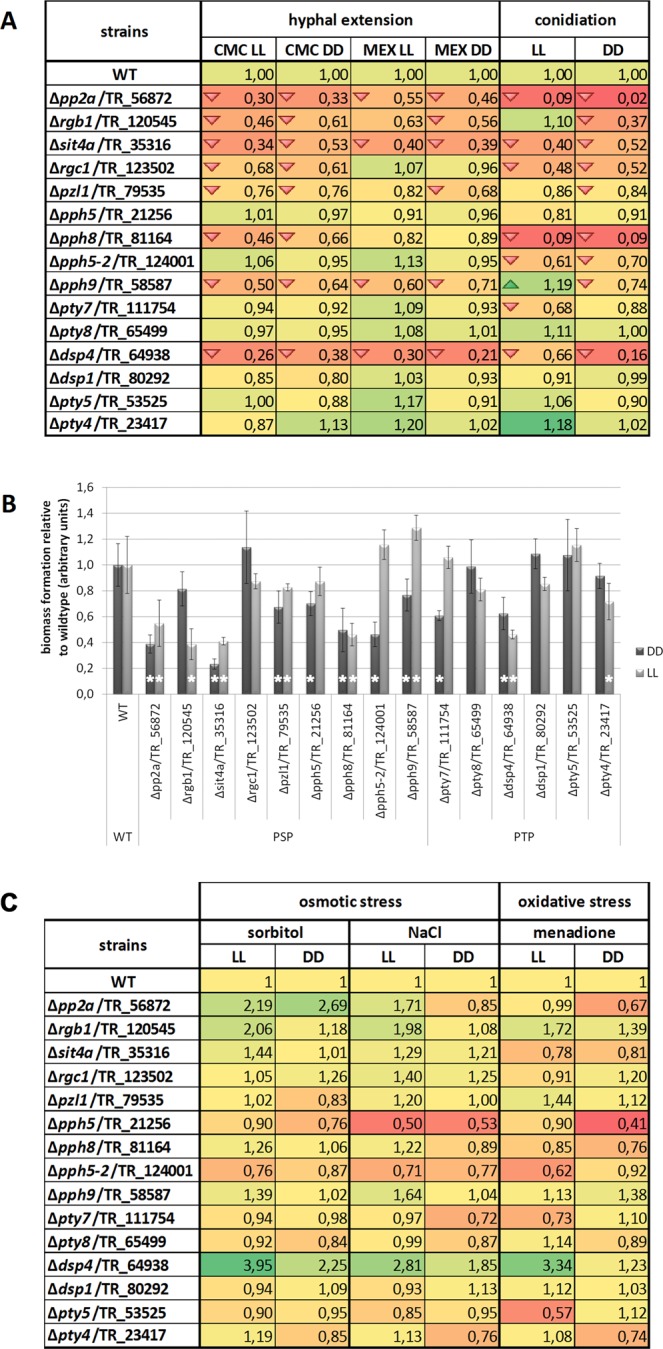
Phenotypic characterization of strains lacking non essential protein phosphatases. (**A**) Analysis of colony diameter in constant light (LL) or constant darkness (DD) upon growth on minimal medium with carboxymethylcellulose (CMC) as carbon source or on malt extract. Conidiation/asexual development was analyzed upon growth on malt extract medium in constant light and constant darkness. Values are shown relative to wildtype. Statistically significant alterations of growth in this panel are marked with a triangle. (**B**) Biomass formation upon growth on minimalmedium with cellulose as carbon source in constant light or constant darkness for 72 hours. Values with statistically significant difference from wildtype under the same condition are marked with an asterisk. Errorbars show standard deviations of three biological replicates. (**C**) Evaluation of responses to osmotic and oxidative stress in constant light and constant darkness. Values were calculated relative to growth of the same strain without the stressor and then the difference was normalized to wildtype. The red background and arrows indicate a decrease and green background and arrows an increase. Details on analysis of statistical significance and p-values are provided in Additional file 2.

For evaluation of the influence of protein phosphatase genes on growth on solid media, we analzyed colony diameters on minimal medium with carboxymethylcellulose (CMC) and on malt extract (MEX) in constant light and constant darkness. Upon growth on CMC, 8 deletion strains showed consistently reduced growth in both light and darkness (Fig. 2A; Additional file 2) and no strain showed a specific light dependent growth defect. Of those, Δ*pp2a*/TR_56872, Δ*sit4a*/TR_35316, Δ*pph9*/TR_58587 and Δ*dsp4*/TR_64938 showed a growth defect on malt extract as well in light and darkness (Fig. 2A), which indicates that these genes are of general relevance for growth in *T. reesei*. In darkness on malt extract, Δ*rgb1*/TR_120545 and also Δ*pzl1*/TR_79535 showed a statistically significant reduction in growth (Fig. 2A). Interestingly, Δ*rgc1*/TR_123502, and Δ*pph8*/TR_81164 exhibited reduced growth only on CMC, suggesting that these protein phosphatases are involved in carbon signaling and potentially cellulase regulation.

The mutant of the homolog of PPH8/TR_81164 in *N. crassa* (NCU4600, PPH-8) also exhibited multiple defects in hyphal growth and asexual sporulation^67^. However, deletion of the homolog in *S. cerevisiae* (PTC2) only resulted in growth defect at high temperatures (37 °C), which is dependent on the HOG pathway^68^.

While deletion of several protein phosphatase genes resulted in decreased growth in light and darkness compared to the wildtype under the same conditions, none of the mutants we investigated showed faster growth than the wildtype under any condition. Moreover, no contrasting phenotypes in light and darkness (increased growth in light and decreased growth in darkness or *vice versa*) were observed.

Investigation of biomass formation in shake flask cultures on minimal medium with cellulose as carbon source clearly confirmed the relevance of *pph8* for growth on cellulose, while *rgc1* was not required for normal growth under these conditions. Besides Δ*pph8*/TR_81164, also Δ*pp2a*/TR_56872 and Δ*sit4a*/TR_35316 showed a strong reduction in growth to about half the biomass formation of the wildtype and 8 more protein phosphatases were found to be relevant for growth on cellulose in liquid culture (Fig. 2B). In part the effect was light dependent. (i. e. for deletion of *pph5*, *pph5-2*, *pty7* and *pty4* significant changes were seen either in light or in darkness). We conclude that the role of these genes changes if the physiology of the fungus changes in response to light.

### Role of protein phosphatases in stress response

Growth and development of microorganism can be affected by several chemicals mimicking environmental stresses. In order to determine whether protein phosphatases are involved in stress response, we performed osmotic and oxidative stress assays with phosphatase mutant strains. To induce osmotic stress we used NaCl and sorbitol which impose ionic stress (NaCl) or, non-ionic stress (sorbitol), respectively^69,70^. Menadione was used as oxidative stress inducing agent.

In general, all protein phosphatase deletion mutants showed statistically significant growth defects under one or more stress conditions in light and/or darkness (Additional file 1; Supplementary Fig. S1). Since several of the deletion strains showed growth defects already without adding stressors, we corrected the growth data (colony diameters) under stress conditions for the growth defects compared to wildtype. We found that particularly *pp2a* and *dsp4* had a negative effect on osmotic stress response because the mutants in these strains showed increased growth in the presence of sorbitol and NaCl, except for Δ*pp2a* in darkness on NaCl where a slightly positive effect on growth was observed (Fig. 2C; Additional file 2). Deletion of *rgb1* or *pph9* resulted in better growth under conditions of osmotic stress particularly in light (Fig. 2C; Additional file 2) although they have a growth defect without stress (Fig. 2A). Lack of eiter *pph5* or *pph5-2* causes no significant alteration in growth compared to the wildtype (Fig. 2A), but defects in response to osmotic stress in light and darkness, particularly in the presence of NaCl (Fig. 2C; Additional File 1).

In agreement with our data, the homolog of PP2A in *N. crassa* (NCU06563, PP2A) was suggested to be involved in osmotic stress response. The deletion mutant Δ*pph8*/TR_81164 showed somewhat altered sensitivity to both osmotic agents both in light and dark conditions. The *S. cerevisiae* homologs of both PP2A/TR_56872 and PPH8/TR_81164 are PPG1 and PTC2, respectively. The proteins PPG1 and PTC2 are required for glycogen accumulation and dephosphorylation of Hog1p, respectively^68,71^. The Hog1p homologue of *T. reesei*, TMK3 (*Trichoderma* MAPkinase 3), was shown to be relevant for cellulase regulation^72^.

The role of the homologs in *N. crassa* (NCU06563, PP2A and NCU04600, PPH-8) and *S. cerevisiae* (PPG1 and PTC2) was suggested to be in regulation of OS-2 dephosphorylation and have important roles in the MAPK signalling cascade^67^. More specifically, Ptc2p was also shown to dephosphorylate Cdc28p in *S. cerevisiae*, likewise functioning with proteins such as RAD53 to regulate DNA damage checkpoint pathways^68,73,74^. Therefore, the role of these protein phosphatases in *T. reesei* and *N. crassa* might be similar.

The regulatory subunit RGB1 is homologous to the gene product of NCU09377 in *N. crassa* (RGB-1), which was shown to be responsible for dephosphorylation by PP2A of the WCC, leading to activation of WC-1^67,75^. Ghosh *et al*. (2014) hypothesized that both the catalytic subunit and the regulatory (RGB-1) subunit of PP2A are able to control expression of the phospho-OS-2 MAPK via the WCC, thus providing additional layers of regulation of phospho-OS-2 MAPK expression^67^. Assuming that the same molecular mechanism occurs in *T. reesei*, in the absence of RGB1/TR_120545, the WCC (BLR-1 and BLR-2) complex would be constantly inactive or at least less active. Therefore, the circadian clock would not function normally, and this is consistent with our results for stress response and conidiation of the mutant Δ*rgb1/TR_*120545, displaying light dependent functions. Compared to wildtype for osmotic stress, the growth of the mutant is only reduced in darkness. Accordingly, a link between light response via BLR1 and BLR2 and stress response was shown previously in *Trichoderma* spp.^43,44^. More generally, the connection between light response and stress response is supported by findings with the photoreceptor ENV1, which impacts activity of the photoreceptor complex. The evolutionarily conserved structure of ENV1 in *Hypocreales* integrates light response with stress response^42^.

The homolog of PZL1 in *S. cerevisiae*, PPZ1, has been characterized as an important regulator of salt and pH homeostasis^76,77^, suggesting a similar role in *T. reesei*. Deletion of the homolog in *N. crassa* (NCU07489, PZL-1) showed resistance to both sorbitol and sodium chloride, confirming the role of this protein in osmotic stress response^67^. In *T. reesei*, a light dependent effect on sorbitol and NaCl was found for *pzl1*, albeit it is less pronounced as that of the aforementioned protein phosphatases.

Upon growth under conditions of oxidative stress, we found different responses in constant light and constant darkness in many cases (Fig. 2C). The strongest effect was observed for *dsp4* in light, for which the severe growth defect on CMC alone was almost alleviated. *Rgb1* was found to have a negative effect on tolerance of oxidative stress in light and darkness. *Pph5* showed a growth defect under oxidative stress in darkness, while *pph5-2* showed a defect in light.

In *N. crassa*, deletion of the homologs for PP2A/TR_56872 (NCU06563, PP2A) and PZL1/TR_79535 (NCU07489, PZL-1) also showed sensitivity to menadione^67^. The homolog of PZL1/TR_79535 in *A. fumigatus* (PHZA) is also involved in oxidative stress tolerance^78^. *T. reesei* PP2A/TR_56872 (and its regulatory subunits) has a positive effect on oxidative stress response in darkness and PZL1/TR_79535 has a negative effect on growth under oxidative stress conditions, particularly in light (Fig. 2C). Consequently, their function in oxidative stress response is conserved in *T. reesei*.

### Protein phosphatases have positive and negative functions in sexual development

We examined sexual development in our protein phosphatase mutants and found that all mutants were able to undergo sexual mating with a fully-fertile strain (no defects in male or female fertility). Hence, the protein phosphatases we tested are not essential for sexual development. Five protein phosphatase mutants showed a delay in fruiting body formation compared to the wildtype (Δ*pp2a*/TR_56872, Δ*dsp4*/TR_64938, Δ*pzl1*/TR_79535, Δ*pph8*/TR_81164 and Δ*sit4a*/TR_35316), while two showed an earlier time-point for fruiting body formation (Δ*pty4*/TR_23417 and Δ*pph5-2*/TR_124001). Of five mutants showing delayed fruiting body formation, three displayed defects in growth (Δ*pp2a*/TR_56872, Δ*dsp4*/TR_64938 and Δ*sit4a*/TR_35316), which implies a somewhat longer time to encounter the mating partner and, therefore, undergo sexual development. Ascospores were discharged for all crosses with mutant strains, although five strains showed a delay in ascospore discharge (Δ*rgb1*/TR_120545, Δ*pp2a*/TR_56872, Δ*dsp4*/TR_64938, Δ*pzl1*/TR_79535, Δ*pph5*/TR_21256 and Δ*sit4a*/TR_35316).

The homolog of PP2A/TR_56872 in *Sordaria macrospora* (SmPP2Ac) was shown to be involved in regulating cell–cell fusion and sexual development as an integral component of the STRIPAK complex^67,79^. Deletion of the homologous gene in *N. crassa* (NCU06563, *pp2A*) resulted in female-sterility^67^. However, since our mutants are in the background strain QM6a, which is female-sterile^80^, we cannot evaluate how the deletion of *pp2a* affects female-fertility in *T. reesei*. Absence of the homolog of DSP4/TR_64938 in *N. crassa* (NCU08158, DSP-4) also exhibited abnormalities during sexual development. Deletion of the homolog of PZL1/TR_79535 in *N. crassa* (NCU07489, PZL-1) only showed an abnormal phenotype during protoperithecia formation (Ghosh *et al*., 2014). Interestingly, deletion of the homolog of PPH8/TR_81164 in *N. crassa* (NCU04600, PPH-8) resulted in unregulated protoperithecia development. PPH-8 was suggested to affect nitrogen sensing and the sexual development pathway. It was hypothesized that PPH-8 regulates protoperithecial development via modulation of the OS-2 MAPK pathway^67^. Concerning the mutant strains that showed an earlier fruiting body formation, the deletion of the homolog of PTY4/TR_23417 in *N. crassa* (NCU09841, PTY-4) exhibited decreased production of protoperithecia. This suggests that PTY-4 may regulate sexual development via OS-2 phosphorylation in *N. crassa*^67^. In *T. reesei*, the absence of PPH5-2/TR_124001 resulted in faster formation of fruiting bodies after crossing. The gene encoding PPH5-2/TR_124001 might be the result of a duplication event in the genome, arising from the gene *pph5*/TR_21256 in *T. reesei*, due to the fact that direct homologs for PPH5-2/TR_124001 are not found in other fungi. The mutant Δ*pph5*/TR_21256 showed a delay in ascospore discharge, which could suggest a necessary cooperation of both proteins (PPH5 and PPH5-2) for a normal sexual development. Also a subfunctionalization of the two paralogs cannot be excluded. However, in *N. crassa* the mutant of the homolog of PPH5/TR_21256 (NCU01767, PPH-5) did not display an abnormal phenotype in sexual development^67^. Finally, the mutant strains of two regulatory subunits (Δ*rgb1*/TR_120545 and Δ*sit4a*/TR_35316) of PP2A proteins showed altered phenotypes during sexual development (delayed discharge of ascospores for both mutants and delayed fruiting body formation for the second). Considering that the catalytic subunits of PP2A are involved in sexual development, the defect observed in the mutants of regulatory subunits is consistent.

### Impacts on conidiation by protein phosphatases

Assessment of asexual development showed that in the deletion strains Δ*pp2a*/TR_56872 and Δ*pph8*/TR_81164 conidiation was almost abolished. An increase in sporulation was only observed in Δ*pph9*/TR_58587 in light (Fig. 2A; Additional file 2). Interestingly, Δ*pph5-2*/TR_124001 showed decreased sporulation in light and darkness. This strain shows earlier fruiting body formation, but a delay in ascospore discharge. Therefore a potential shift in preference between asexual and sexual development by PPH5-2 warrants further investigations.

The mutant of the homolog to PPH8/TR_81164 in *N. crassa* (NCU04600, PPH-8) also showed defects in conidiation^67^. Similarly, a mutant of the homologs to PP2A/TR_56872 (NCU06563, PP2A) also showed a defect during asexual development, which is in line with our findings. Accordingly, also the PP2A regulatory subunit mutants (Δ*rgb1*/TR_120545 and Δ*sit4a*/TR_35316) show conidiation defects. However, deletion of the homolog to DSP4/TR_64938, which affects conidiation, did not cause any adverse effects on conidiation in *N. crassa* but deletion of the homologs in *S. cerevisiae* (YVH1), and *M.oryzae* (MoYvh1) resulted in reduced growth and sporulation (yeast) and lower conidia production (*M. oryzae*)^81^.

There are also PPs involved in asexual development in *N. crassa* that show no significant effect in *T. reesei* (PPH5/TR_21256-NCU01767 (PPH-5), DSP1/TR_80292-NCU03426 (DSP-1) and PTY5/TR_53525-NCU01010 (PTY-5)) revealing a different function of those PPs in both fungi^67^. Importantly, three mutant strains (Δ*rgb1*/TR_120545, Δ*dsp4*/TR_64938 and Δ*pph9*/TR_58587) exhibited reduced conidiation in constant darkness but not in constant light compared to the wildtype (Fig. 2A). These genes are regulated during different stages of conidiation^82^. Thus, *rgb1, dsp4* and *pph9* are not generally involved in regulation of conidiation. Instead their importance for conidiation depends on whether the fungus grows in light or in darkness.

### Pty4, Pzl1 and Dsp1 regulate protease production in a light dependent manner

Fungal proteases are a major bottleneck in industry for efficient production of proteins of interest^83,84^. Although in microbial production systems the genes encoding proteases could be deleted^85^, the large number of genes encoding proteases in fungi makes it impossible to remove them all. A strain harboring the deletions of seven proteases was reported to support stable expression levels of therapeutic proteins^85^. Nevertheless, there is limited knowledge about the molecular regulation by which proteases are produced.

**Figure 3:**
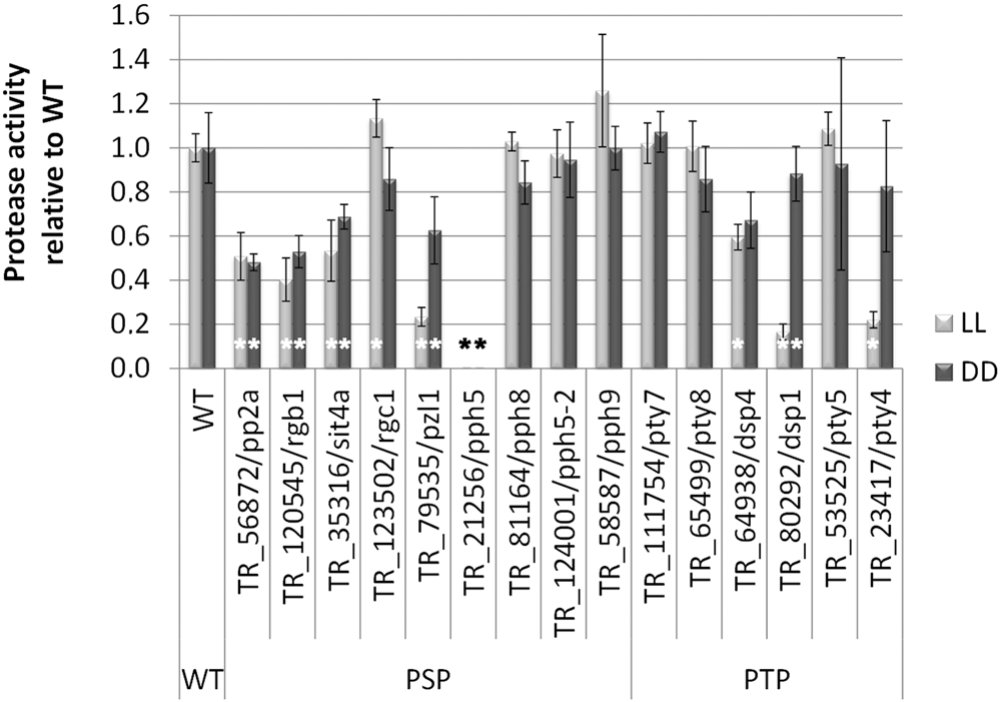
Analysis of protease production. Strains were grown in constant light (LL) or constant darkness (DD). Genes deleted in the respective strains are shown on the x-axis. Asterisks indicate statistically significant different protease production compared to wildtype under the same conditions. Strains were grown on TSA media amended with 10% (w/v) powdered milk at 28 °C after 2 days. Calculations are based on the ratio halo/mycelium and normalized to wildtype in light and darkness correspondingly. Errorbars show standard deviations of three biological replicates. Details on analysis of statistical significance and p-values are provided in Additional file 2.

We investigated the proteolytic activity secreted by our deletion strains under constant light or dark conditions (Fig. 3). Δ*pp2a*/TR_56872, Δ*rgb1*/TR_120545, Δ*sit4a*/TR_35316, Δ*pzl1*/TR_79535, Δ*pph5*/TR_21256 and Δ*dsp4*/TR_64938 showed decreased protease activity in light and darkness. Δ*pty4*/TR_23417 and Δ*dsp1*/TR_80292 showed strongly decreased protease activity specifically in light.

Three mutant strains (Δ*pty4*/TR_23417, Δ*pzl1*/TR_79535 and Δ*dsp1*/TR_80292) showed a more pronounced decrease of proteases under constant light compared to darkness. Although there is a difference in protease production in the wildtype between light and darkness (slightly decreased in darkness), the observed result for the deletion strains exceeds these differences and is therefore specific for the deleted genes.

### A relevance for protein phosphatases in cellulase regulation

**Figure 4:**
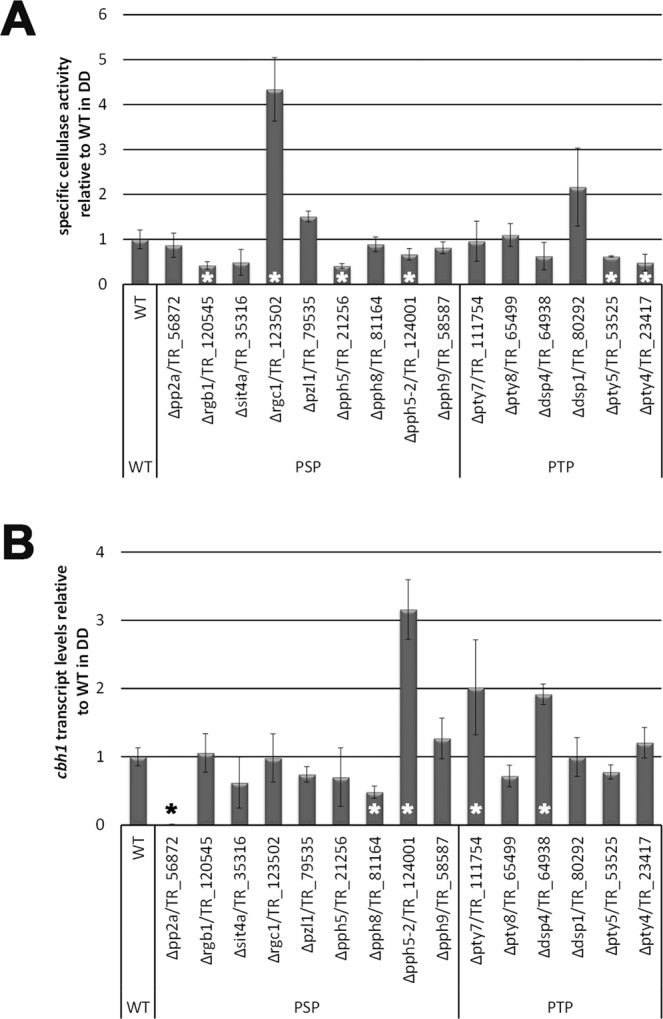
Analysis of the influence of PPs on cellulase regulation. (**A**) Specific cellulase activity, i. e. cellulase activity in the culture filtrate relative to produced biomass. Strains were grown on Mandels Andreotti minimal medium with cellulose as carbon source in constant darkness. Errorbars show standard deviations of three biological replicates. Details on analysis of statistical significance and p-values for specific cellulase activity are provided in supplementary file 2. (**B**) Transcript abundance for the major cellulase gene *cbh1* analyzed in strains grown as described for (**A**). For statistical significance, a p-value threshold of 0.05 was applied and three biological and three technical replicates were considered.

We studied the specific cellulase activity upon growth with cellulose as carbon source in constant darkness reflecting conditions in industrial fermenters. We found clearly increased specific cellulase activity compared to wildtype for Δ*rgc1*/TR_123502 and a positive trend for Δ*dsp1*/TR_80292 (Fig. 4A). Δ*rgc1*/TR_123502 exhibited a defect in hyphal growth (as reflected in achieved colony diameter) in light and darkness only when growing on cellulose, but not on malt extract (Fig. 2), indicating more efficient cellulase production at the expense of biomass formation. Therefore, RGC1 might be involved in negative regulation on cellulase production. In contrast, Δ*rgb1*/TR_120545, Δ*pph5*/TR_21256, Δ*pph5-2*/TR_124001, Δ*pty4*/TR_23417 and Δ*pty5*/TR_53525 showed decreased specific cellulase activity and in Δ*sit4a*/TR_35316 we found a negative trend (Fig. 4A). This reduction suggests that these PPs positively regulate production of plant cell wall degrading enzymes.

Analysis of transcript levels of the major cellulase gene *cbh1* showed that the increased specific cellulase activity of Δ*rgc1*/TR_123502 and the positive trend in Δ*dsp1*/TR_80292 (Fig. 4B) do not correspond with an increased *cbh1* transcription level or altered biomass formation on cellulose (Fig. 2B). Hence, posttranscriptional regulation as shown recently^64^ potentially contributes to this regulation. Alternatively, other enzymes regulated by these PPs could be responsible for this effect. *cbh1* transcript levels were decreased compared to wildtype in the deletion strains Δ*pp2a*/TR_56872 and Δ*pph8*/TR_81164. In Δ*pp2a*/TR_56872 biomass production on cellulose was reduced (Fig. 2B), which is in agreement with lower *cbh1* transcript levels. However, the residual growth and activity indicate that cellulase degradation was not totally abolished. Δ*dsp4*/TR_64938, Δ*pty7*/TR_111754 and *Δpph5-2*/TR_124001 showed higher transcript levels of *cbh1* compared to the wildtype, although this result does again not correlate with the specific cellulase activity levels. Interestingly, *dsp4*/TR_64938 is located within a genomic cluster of genes regulated in an induction specific manner (cluster 20^64^). We conclude that protein phosphatases play important roles in the posttranscriptional section of cellulase regulation, which was shown recently^64^ and/or coordinated regulation of CAZymes for efficient cellulose degradation. This regulation likely involves (de) activation and modulation of protein stability of regulatory proteins, which is often dependent on the phosphorylation status.

### Protein phosphatases impact secondary metabolism

*Trichoderma* spp. produce a great diversity of secondary metabolites^86^. In *T. reesei*, recent studies indicate a regulatory interconnection between carbon and secondary metabolism with the carbon catabolite repressor CRE1^87^ and the transcription factor XPP1^88^ as most important dual regulators. To study if protein phosphatases are involved in this regulation, secretion patterns of secondary metabolites in the deletion strains were analyzed. For that purpose, semi-quantitative high performance thin layer chromatography (HPTLC) was applied.

**Figure 5:**
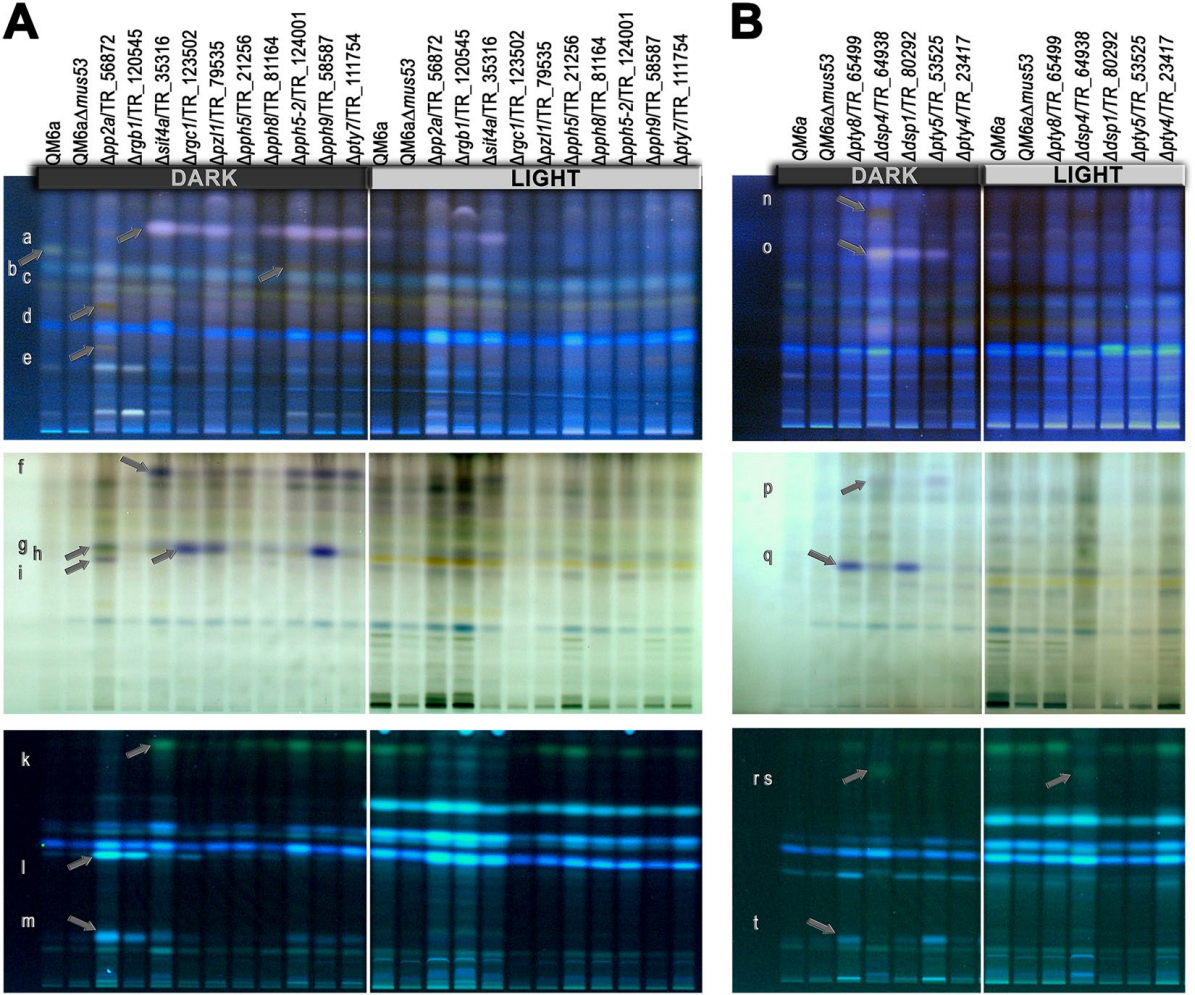
Analysis of secreted secondary metabolite patterns. (**A**) Strains lacking PSPs or (**B**) strains lacking PTPs were grown on minimal medium with cellulose as carbon source in constant light or constant darkness. Amounts of supernatants applied for analysis were normalized to equal amounts of biomass produced by the respective strain. Letters at the edge of the figure refer to an arrow indicated an altered band to the right of the letter. Visualizations shown are derivatized/remission at 366 nm (upper panel), derivatized/transmission (middle panel) and developed, 366 nm (lower panel).

Altered secondary metabolite patterns were observed for several deletion strains (Fig. 5). Over all, we observed striking differences in secondary metabolite patterns between light and darkness. The differences between strains were more pronounced in darkness than in light. Δ*pp2a*/TR_56872 as well as the deletion strain for the putative regulatory subunit for PP2A, Δ*rgb1*/TR_120545 showed an increase in secondary metabolite production in light and darkness. These two strains also showed additional bands compared to the wildtype (Fig. 5A bands l and m), some of which were only visible in Δ*pp2a* (Fig. 5A, bands d, e, g and i), but not Δ*rgb1*. Hence, the secondary metabolite patterns of the two strains are not entirely similar on the different visualizations supporting that RGB1 acts as a regulatory subunit to PP2a, but has other functions as well. In the remaining strains except for Δ*pph5*/TR_21256, an additional band appeared (Fig. 5A band a). Δ*pph5-2*/TR_124001 did produce this additional band and moreover an additional band that is not present in the other strains (Fig. 5A band c), reflecting functional differences between PPH5 and PPH5-2.

Bands f and k, which also appeared in these strains, were hardly detectable in Δ*rgc1*/TR_123502, but are present in Δ*pph5*/TR_21256 (Fig. 5A). A band present in the wildtype samples was not detected in most mutant samples except for a weak appearance in Δ*pph5*/TR_21256 (Fig. 5A band b). Band h is enhanced in Δ*rgc1*/TR_123502, Δ*pzl1*/TR_79535 and Δ*pph9*/TR_58587 (Fig. 5A). The lower signal strength for Δ*rgc1*/TR_123502 in light indicates less secondary metabolites to be produced (Fig. 5A). Interestingly, the homologue of *pzl1* in *A. fumigatus* (*ppzA*) is involved in regulation of secondary metabolism as well, particularly in connection to iron starvation and siderophore production, with deletion of *ppzA* leading to loss of pathogenicity of *A. fumigatus*^89^.

Δ*dsp4*/TR_64938 showed increased amounts of secreted secondary metabolites with additional bands (Fig. 5B bands n, o, p, r and s) not only in darkness but also in light. Band o is also additionally present in Δ*dsp1*/TR_80292 and Δ*pty5*/TR_53525 (Fig. 5B). Δ*pty8*/TR_65499 and Δ*pty5*/TR_53525 showed a further enhanced band in darkness compared to wildtype (Fig. 5B, band t).

In summary, our screening for secondary metabolite patterns showed a clear influence of many protein phosphatases on secondary metabolite production. In some cases individual bands (which could represent more than one compound) are concerned, but we also found an overall effect on the amount of secondary metabolites produced for some strains. The effects on individual compounds were mostly limited to darkness. Consequently, protein phosphatases contribute to the physiological effects of light on *T. reesei* as compared to growth in darkness, which are mediated by signaling pathways also by regulation of secondary metabolism.

### Functional clustering of protein phosphatases

**Figure 6:**
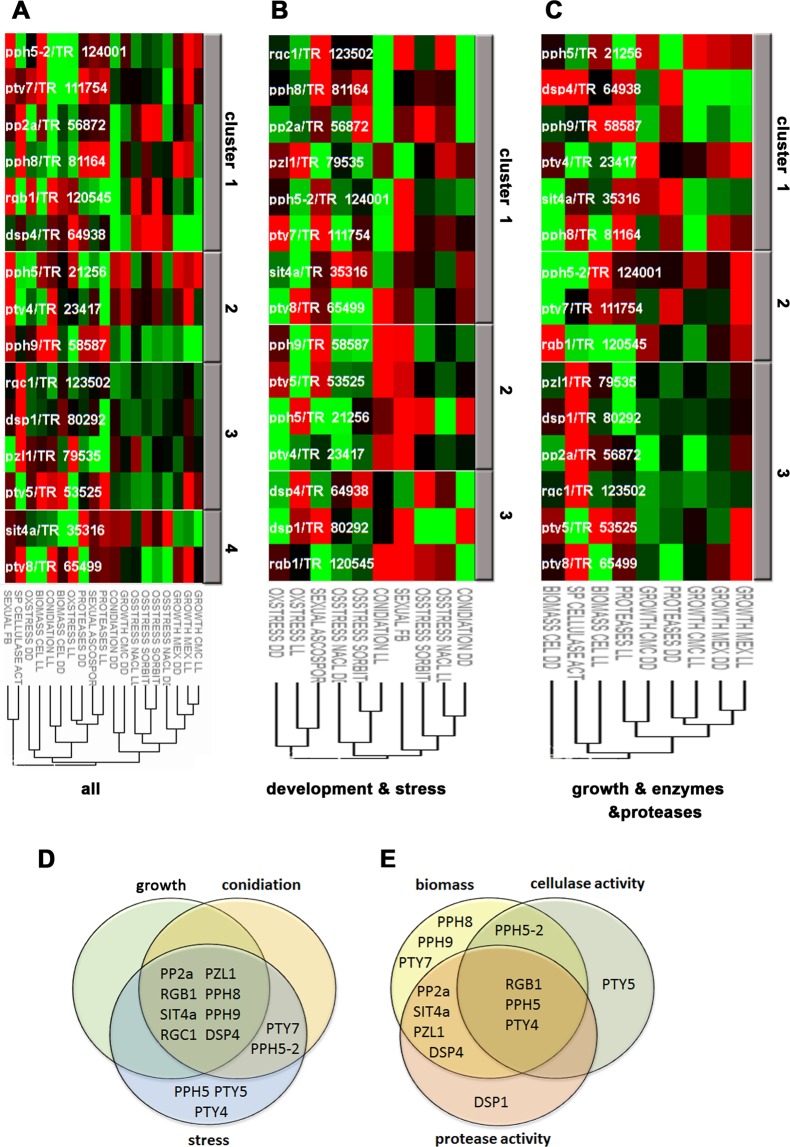
Hierarchical clustering of phenotypic characterization. (**A**) Clustering of all analyses. (**B**) Clustering of results for analysis of development and stress response. (**C**) Clustering of results for growth/hyphal extension on malt extract medium (MEX) or carboxymethylcellulose (CMC), biomass production on cellulose, protease production and specific cellulase activity. DD: constant darkness, LL: constant light. Details on statistical analysis of results are provided in Additional file 2.

Based on the phenotypic traits we investigated, we performed hierarchical clustering of effects related to wildtype in our deletion strains. This analysis on the one hand provided us with information on common functions of individual protein phosphatases. On the other hand the clustering revealed phenotypical traits affected in the same way by several deletions, which indicates that the underlying regulatory pathways are connected. Assessing all traits, we found clustering of growth and osmotic stress together, but separate from development and oxidative stress (Fig. 6A). The results described above showed phenotypic similarities for some strains, but alterations of such similarities depended on the conditions, indicating the individual functions in the regulatory network of phosphorylation for all investigated genes.

Separate clustering of phenotypes in stress response and development (Fig. 6B) as well as of growth, enzyme production and protease secretion (Fig. 6C) showed that deletion of *pp2a* or its putative regulatory subunit *rgb1* causes phenotypes that are too dissimilar to cluster together. Δ*pph5*/TR_21256 and Δ*pph5-2*/TR_124001 did not appear in a common cluster, which supports the hypothesis that *pph5-2* assumed complementary functions to *pph5* and that a subfunctionalization of these paralogs has happened. Moreover, we could not detect sets of protein phosphatases acting similarly across conditions (Fig. 6A–C). Although this was unexpected, it highlights the complex regulation of phosphorylation states by protein phosphatases and their regulatory subunits.

With the aim to put the functions of the non essential phosphatases in context, we investigated whether the genomic locations of the encoding genes are within or close to known functional clusters. We found no PP encoding genes within CAZyme clusters^64,90^ or in light regulated clusters on cellulose^55,64^. However, *dsp4*/TR_64938 is located within cluster 20 of induction associated genes (supplemental dataset 4 of reference^56^), which also comprises *egl5/cel45a* encoding endoglucanase 5^64^. *dsp4* shows a significant decrease in biomass formation upon growth on cellulose in light and darkness, which suggests a role in support of normal growth on cellulose.

## Conclusions

Protein phosphatases constitute, together with protein kinases, key players as the currency of signaling in the cell. The set of deletion strains analyzed in this article showed that only some of them displayed defects in growth and development compared to wildtype. In contrast, most of the strains showed alterations in stress response. With respect to enzyme production, several strains showed decreased specific cellulase activity and protease production, albeit no correlation of increased cellulase production and decreased protease activity or *vice versa* was found. In many cases the defect caused by deletion of individual PPs concerned more than one functional pathway (Fig. 6D,E). Considering the interconnection between cellulase production and secondary metabolism that was postulated recently^87,88^, we found several strains with alterations in both pathways. For example, deletion of *rgc1* causes an increase in specific cellulase activity (Fig. 4A) as well as appearance of additional secondary metabolite bands (Fig. 5A, bands a and g). Band c (Fig. 5A) appears in ∆*pph5-2*/TR_124001, which shows decreased cellulase activity. In case of *pp2a* we saw several additional bands (Fig. 5A bands d, e, g, i, l and m) and a decrease in *cbh1* transcript levels, while cellulase activity appears to be complemented by other cellulolytic enzymes. Consequently, the connection between secondary metabolism and enzyme formation turns out to be a broad mechanism not limited to one or a few transcription factors or to a cluster. Rather, this connection appears as a general phenomenon, where detection of a regulatory impact on cellulases has to be expected to include also an influence on secondary metabolism.

Our results confirm a central role of PP2A catalytic and regulatory subunits for primary and secondary metabolism. The homolog of PP2A/TR_56872 in *N. crassa* (PP2A/NCU06563) also showed several defects in growth, development and stress response^67^. Moreover, in *N. crassa* PP2A is involved in the OS-2 pathway, confirming the involvement in stress response^67^. A homolog of the putative regulatory subunit of PP2A, RGB1/TR_120545 in *S. cerevisiae* Cdc55 is involved in Rho signaling, that regulates cell growth and stress response^91^. The homolog in *N. crassa* (RGB-1/NCU09377) is responsible for dephosphorylation of WCC^75^ and the WCC controls phospho-OS-2^92^. In *T. reesei*, the mutant Δ*rgb1*/TR_120545 shows different phenotypes depending on the light conditions, which is in agreement with a role in light dependent physiology. It remains to be determined whether PP2A/TR_56872 and RGB1/TR_120545 have a common influence on the OS-2 pathway, on Rho signaling and on regulation of the circadian clock in *T. reesei*.

Analysis of specific cellulase activity showed that PPs influence the production of cellulases. Involvement of protein phosphatases in cellulase production was previously studied in *A. nidulans*. In this respect, homologs of PP2A/TR_56872 (Ppf1A/AN0164), PZL1/TR_79535 (PpzA/AN3793), PPH9/TR_58587 (PtcA/AN6892) and PTY4/TR_23417 (LtpA/AN10570), defined as non-essential protein phosphatases, were described to be involved in cellulase production in *A. nidulans*^27^. In *T. reesei* deletions of these protein phosphatases caused decreased growth on cellulose in Δ*pp2a*/TR_56872, Δ*pty4*/TR_23417 and Δ*pzl1*/TR_79535. Therefore, the role of those protein phosphatases is consistent between *A. nidulans* and *T. reesei* with the exception of *pph9*. Besides their role in endocellulase production, the function of these proteins was inferred via homology to *S. cerevisiae* if not characterized already in *Aspergilli*^27^. Among them, one has a role in the cell cycle, which is homologous to a low-molecular-weight protein tyrosine phosphatase (LtpA/AN10570, homologue: PTY4/TR_23417). Furthermore, a homolog to one PP has a role in MAPK regulation (PtcA/AN6892, PPH9/TR_58587)^93^. A PP2A homologue in *S. cerevisiae*, Ppg1p (homologous to Ppg1A/AN0164 in *A. nidulans* and PP2A/TR_56872 in *T. reesei*), is involved in glycogen accumulation^27^. Tap42 (TAP42/YMR028W) and Sit4 (SIT4/YDL047W homologous to the PSP TNG/TR_48910) are associated with Ppg1p in *S. cerevisiae*, being involved in TOR signaling^27^.

Δ*dsp1*/TR_80292 and Δ*rgc1*/TR_123502 showed an increased specific cellulase activity compared to wildtype. Δ*rgc1*/TR_123502 showed a growth defect in liquid culture on cellulose. However, decreased growth was also detected on plates with carboxymethylcellulose and maltextract, indicating that the effect on growth is general and is not caused by the altered cellulase production. Interestingly, RGC1/TR_123502 was recently shown to be significantly phosphorylated upon induction with sophorose^11^, and this protein harbors a starch/glycogen-binding module. Taken together, these results suggest that the regulatory subunit RGC1/TR_123502 is required for normal growth and negatively regulates cellulase production in *T. reesei*.

Bidirectional best hit analysis and phylogenetic analysis indicates that PPH5-2/TR_124001 arose from a duplication event of *pph5*/TR_21256. PPH5-2/TR_124001 is only found in *Trichoderma spp*.^46^. In the genome, *pph5* and *pph5-2* are both located on chromosome 5, but not close to each other^94^. Transcriptome data showed that transcript levels of *pph5-2*/TR_124001 are decreased upon growth on cellulose compared to glucose, glycerol or lactose while *pph5*/TR_21256 shows an opposite trend in regulation^64^. Hence, both genes displayed opposite transcription patterns.

Our analyses clearly showed different phenotypic patterns for Δ*pph5*/TR_21256 and Δ*pph5-2*/TR_124001 in many cases. However, the homolog in *N. crassa* (NCU01767, PPH-5) is required for normal growth of basal hyphae and asexual development^67^, which is not the case for the *T. reesei* homologues. Only Δ*pph5-2*/TR_124001 shows a significant decrease in conidiation. The *S. cerevisiae* homologue PTC5 is also required for normal vegetative growth^95^. Hence, PPH5 and PPH5-2 assume distinct functions in *T. reesei*, which are not entirely conserved in *N. crassa* and *S. cerevisiae*.

In summary, we demonstrated a broad diversity of functions of protein phosphatases in *T. reesei*, organized in a complex network of target pathways, which is in many cases specific for a given light condition and a carbon source.

## Methods

### Microbial strains and culture conditions

*Trichoderma reesei* (syn. *Hypocrea jecorina*) strain QM6a (ATCC13631) and the non-homologous end joining (NHEJ) deficient strain QM6aΔ*tmus53*^96^ were used throughout this study. For genotypes of the strains used and prepared for this study see Table 1. All analyses done in constant darkness including harvesting mycelia were done with red safety light (darkroom lamp, Dr. Fischer 230 V, red, E27, 15 W).

**Table 1:** Strains used in this study.

Strain	Characteristics	Source/Reference
QM6a	Wildtype (ATCC13631)	^90^
QM6aΔ*tmus53*		^96^
FF1	Female fertile derivative of QM6a Mat 1-1	^104^
Δ*pp2a*	QM6aΔ*tmus53*Δ56872::*hph*^+^	This study
Δ*rgb1*	QM6aΔ*tmus53*Δ120545::*hph*^+^	This study
Δ*sit4a*	QM6aΔ*tmus53*Δ35316::*hph*^+^	This study
Δ*rgc1*	QM6aΔ*tmus53*Δ123502::*hph*^+^	This study
Δ*pzl1*	QM6aΔ*tmus53*Δ79535::*hph*^+^	This study
Δ*pph5*	QM6aΔ*tmus53*Δ21256::*hph*^+^	This study
Δ*pph8*	QM6aΔ*tmus53*Δ81164::*hph*^+^	This study
Δ*pph5-2*	QM6aΔ*tmus53*Δ124001::*hph*^+^	This study
Δ*pph9*	QM6aΔ*tmus53*Δ58587::*hph*^+^	This study
Δ*pty7*	QM6aΔ*tmus53*Δ111754::*hph*^+^	This study
Δ*pty8*	QM6aΔ*tmus53*Δ65499::*hph*^+^	This study
Δ*dsp4*	QM6aΔ*tmus53*Δ64938::*hph*^+^	This study
Δ*dsp1*	QM6aΔ*tmus53*Δ80292::*hph*^+^	This study
Δ*pty5*	QM6aΔ*tmus53*Δ53525::*hph*^+^	This study
Δ*pty4*	QM6aΔ*tmus53*Δ23417::*hph*^+^	This study

### Generation of deletion strains

The target phosphatase genes for deletion in *T. reesei* were annotated previously^46^. For deletion vector construction of protein phosphatase genes in *T. reesei*, the method of yeast mediated recombination cloning into the plasmid pRS426 with the hygromycin resistance cassette was used as described previously^97,98^. Deletion cassettes (around 3500 bp) were amplified by PCR using pre-designed primers^98^ and transformed to QM6aΔ*tmus53* strain by the polyethylene glycol-mediated protoplast transformation method^99,100^. Selection of colonies was performed on 3% (w/v) malt extract agar plates containing 100 μg ml^−1^ hygromycin B (Roth, Karlsruhe, Germany). After two rounds of single spore isolation, stable transformants were obtained. Deletion strains were confirmed by PCR using primers binding within the deleted region as described previously using the screening primers specified in^98^. Only if no amplicon was obtained with the screening primers binding within the deleted region, strains were considered for further analysis. The copy number of the deletion cassettes integrated in the deletion strains was analyzed using RT-qPCR as described previously^56^. For subsequent analyses, up to three different transformants (if available) were analyzed per deletion to exclude random effects due to the transformation process. Additionally, copy numbers of the deletion cassettes were analzyed to assess the potential of off target effects. Results for strains bearing more than one copy (Additional file 1; Supplementary Table S2) of the deletion cassette were only considered if consistent with the other strains.

### Bioinformatic analysis

Transcriptome data used in this study were obtained and analyzed previously^55,56,64,66^ and are deposited at NCBI Gene Expression Omnibus with accession numbers GSE36448, GSM683732, 683733, 683734 and 683735. Hierarchical clustering was done using the open source software HCE 3.5^101^ with default settings using the Pearson correlation algorithm.

Statistical significance of differences to the wildtype was evaluated using the open source software PSPP 1.0.1 (version August 2017). Statistical data for the phenotypic assays shown in Figs 2, 3 and 4 are provided in Additional file 2.

### Analysis of growth

Deletion strains were grown on plates with 3% (w/v) malt extract (Merck, Darmstadt, Germany) or Mandels-Andreotti medium^102^ containing 1% (w/v) carboxymethylcellulose (CMC) as carbon source, for 2-4 days at 28 °C in constant light (≈1800 Lx) or constant darkness. The growth in constant darkness was analyzed after two days by measuring the colony diameter. The mutant strains were inoculated in the center of the plate and colony diameter reflecting hyphal extension was measured every 24 hours for two to four days. QM6aΔ*tmus53* was used as control for every set. At least three biological replicates were analyzed for every strain.

### Evaluation of development

For evaluation of sexual development, crossing experiments were prepared on 3% (w/v) malt extract at 22 °C under daylight conditions (cycles of 12 h light-12 h dark), which is the preferred condition for sexual development to happen^80,103^. Briefly, strains were inoculated on opposite sides of petri dishes and the female fertile strain FF1^66,104^ was used as crossing partner. Fruiting body formation, size and quantity of fruiting bodies, as well as the ability for ascospore discharge and corresponding quantity were considered. QM6aΔ*tmus53* was used as control.

For assessment of asexual development, deletion strains were grown on 3% (w/v) malt extract for eight days at 28 °C either in constant light (≈1800 Lx) or constant darkness. Three plugs per plate from three independent plates were cut and spores were suspended in 4 ml of spore solution (0.8% (w/v) NaCl with 0.05% (v/v) Tween 80 (Merck, Darmstadt, Germany)). The amount of spores was determined by measurement of the optical density with a spectrophotometer (OD at 600 nm, spectrophotometer from Hitachi, model U-2900) using a standard curve based on microscopic counting. QM6aΔ*tmus53* was used as control for every set. At least three biological replicates were analyzed per strain.

### Osmotic and oxidative stress assays

For assessment of oxidative stress response, strains were cultivated on Mandels-Andreotti minimal medium with carboxymethylcellulose (CMC) (Sigma Aldrich, St. Louis, USA) (1% w/v) as carbon source, supplemented with 0.25 mM menadione (Sigma, St. Louis, USA). To analyze osmotic stress response, mutant strains were grown on solid Mandels-Andreotti minimal medium containing 1% (w/v) carboxymethylcellulose (CMC) as carbon source, supplemented with 1 M NaCl (Merck, Darmstadt, Germany) or 1 M D-Sorbitol (Sigma Aldrich, St. Louis, USA).

Growth was monitored for three days either in constant light (≈1800 Lx) or constant darkness. Mutant strains were inoculated in the center of the plate and colony diameters were measured every 24 hours for three days at 28 °C. QM6aΔ*tmus53* was used as control for every set. At least three biological replicates were analyzed per strain.

### Analysis of protease activity

Protease secretion was determined after growing the strains on 1/5 TSA (15 g/L Tryptone (Roth, Karlsruhe, Germany), 5 g/L Soytone (Merck, Darmstadt, Germany), 5 g/L NaCl, 15 g/L Agar-Agar (Roth, Karlsruhe, Germany)) amended with 10% (w/v) powdered milk (Roth, Karlsruhe, Germany) at 28 °C in constant darkness or constant light (1800 lux). The mutant strains were inoculated in the center of the plate and colony diameter as well as halo diameter were measured every 24 hours for 2 days at 28 °C. A halo appearing around the inoculated area is indicative for exoprotease activity^105^. Calculations are based on the ratio halo/mycelium. QM6aΔ*tmus53* was used as control for every set. At least three biological replicates were analyzed per strain.

### Biomass determination

For analysis of biomass production in the presence of cellulose, strains were grown on Mandels-Andreotti minimal medium with 1% (w/v) microcrystalline cellulose (Alfa Aesar, Karlsruhe, Germany) in constant light and constant darkness as described previously^106^. Cultivations were done in triplicates. In all cases, plates for inoculation were grown in constant darkness for 14 days to avoid interference by circadian rhythmicity, and harvesting was done after 72 hours of growth either in light or under a red safety light (darkroom lamp, Dr. Fischer 230 V, red, E27, 15 W) for cultures grown in constant darkness. QM6aΔ*tmus53* was used as control for every set. At least three biological replicates were analyzed for every strain.

Due to the insoluble cellulose present in the culture, the indirect method of biomass determination was used as described previously^107^. Briefly, mycelium was snap frozen in liquid nitrogen and ground in a Retsch Mill (Retsch MM301, Haan, Germany) in pre-cooled jars for 30 seconds with an oscillation frequency of 30 Hz and transferred to tubes containing 5 ml 0.1 M NaOH (Merck, Darmstadt, Germany). Samples were sonicated with 70% amplitude three times for 30 seconds and incubated at room temperature for 3 h. After centrifugation for 10 minutes at full speed, the protein content of the supernatant as a corresponding measure of biomass was measured by the Bradford method (Roti-Quant, Roth, Karlsruhe, Germany). At least three biological with two technical replicates were analyzed.

### Cellulase activity

Strains were grown in liquid medium as described above, and endo-1,4-β-d-glucanase activity from culture filtrates harvested after 72 hours was measured using the azo-CMC-cellulose kit (S-ACMC-L, Megazyme, Wicklow, Ireland) according to the manufacturer’s instructions. For specific cellulase activity, enzyme activity was correlated to biomass. At least three biological with two technical replicates were analyzed.

### Isolation of total RNA and RT-qPCR

For isolation of RNA, mycelia grown for 72 hours with cellulose as carbon source in constant light or constant darkness as described above were used. Isolation and quality control of total RNA as well as RT-qPCR were performed as described previously, with the ribosomal gene *l6e* as reference gene^108^. Primers used are taken from^56^. RT-qPCR was performed for three biological replicates with three technical replicates each. Data analysis was done using the software package CFX Maestro (Biorad, Hercules, CA, USA).

### Analysis of secondary metabolites production

High performance thin-layer chromatography (HPTLC) analysis was performed as described earlier^104,109–111^ with modifications. Supernatants from the cultures used for analysis of specific cellulase activity were used. The volume of supernatants used for each sample corresponds to equal amounts of biomass. One volume of supernatant was mixed with 1 volume of acetone (1:1; v/v) (Roth, Karlsruhe, Germany). After shaking for 1 hour (400 rpm at RT), 1 volume of chloroform (Roth, Karlsruhe, Germany) was added (1:1 v/v). After shaking again for 30 minutes (400 rpm at RT) and centrifugation for 1 minute at 500 g, the organic phase (chloroform-lower phase) was transferred to a conical tube. After complete evaporation of the solvent, the samples were resuspended in 140 μl chloroform and 9 μl were spotted on a HPTLC plate (HPTLC silica gel 60 F254s, Merck 1.15696.0001) using the CAMAG Automatic TLC sampler 4 (CAMAG, Muttenz, Switzerland). This amount was chosen in order to detect smaller changes in metabolite patterns without overloading the plate. Separation was performed in a saturated twin trough chamber with chloroform:trifluoroacetic acid 7:1 (v/v). The plates were analyzed under ultraviolet light (254 nm and 366 nm) using a CAMAG visualizer (CAMAG). Additionally, the plates were derivatized with p-anisaldehyde:sulfuric acid and evaluated again with white and ultraviolet light. Results were visualized using the software visionCATS 1.4.14017.1 (CAMAG).

## Supplementary information

Additional file 1

Additional file 2

Additional file 3

## Data Availability

All data generated during this study are included in this published article and its supplementary file. GenBank Accession numbers for datasets analyzed for this study are given in Methods and described in the respective cited articles.
