# Supplementary material for: Protein phosphatases regulate growth, development, cellulases and secondary metabolism in Trichoderma reesei
Source: Sci Rep. 2019 Jul 29;9:10995. doi: 10.1038/s41598-019-47421-z (PMC6662751; doi:10.1038/s41598-019-47421-z)
Supplement: Supplementary file 1 — Additional file 1 [file 41598_2019_47421_MOESM1_ESM.pdf]

Protein phosphatases regulate growth, development, cellulases and secondary metabolism in *Trichoderma reesei*

Aroa Rodriguez Iglesias and Monika Schmoll*

Austrian Institute of Technology GmbH, Health & Environment, Bioresources, Konrad-Lorenz-Straße 24, 3430 Tulln, AUSTRIA

Supplementary material

Table S1. Comparison of mutants available in *T. reesei* and *N. crassa*

Gene	Protein	Grou p	Subgroup	*N. crassa* homolog	KO in *N. crassa*	KO in *T. reesei*
pph5	21256	PSP	PP2C	NCU01767	HO	VA
ppp1/p	120722	PSP	PP1	NCU00043	HE	IV
pph8	81164	PSP	PP2C	NCU04600	HO	VA
pzl1	79535	PSP	PP1	NCU07489	HO	VA
pph1	74884	PSP	PP2A	NCU06630	HE	IV
cna1/p	59944	PSP	PP2B	NCU03804	HE	IV
pph9	58587	PSP	PP2C	NCU00434	NA	VA
pp2A	56872	PSP	PP2A	NCU06563	HO	VA
ppt1	52144	PSP	PP5	NCU01433	HO	IV
tng	48910	PSP		NCU03436	HO	IV
pph5-2	124001	PSP	PP2C	-	-	VA
pph4	55868	PSP		NCU08301	HO	VA
pph6	74030	PSP	PP2C	NCU03495	HO	IV
pty4	23417	PTP	LMW-PTP	NCU09841	HO	VA
pty7	111754	PTP	PTP	NCU06969	HO	VA
ptpla	105055	PTP	PTP	NCU08985	HE	IV
pty2	77689	PTP	PTP	NCU02257	HO	VA
pty8	65499	PTP	PTP	NCU01889	HO	VA
pty5	53525	PTP	PTP	NCU01010	HO	VA
div12	53004	PTP	CDC25	NCU02496	HE	IV
pty6	41428	PTP	PTP	NCU03333	HO	IV
pty9	5737	PTP	PTP	-	-	VA
pty10	121175	PTP	PTP	-	-	IV
dsp4	64938	PTP	PTP	NCU08158	HO	VA
dsp1	80292	PTP	PTP	NCU03426	HO	VA
dsp2	119697	PTP	PTP	NCU06252	HO	IV
pty3	25159	PTP	PTP	NCU05364	HO	IV
cdc14	27406	PTP	DSP	NCU03246	HO	IV
rgc1	123502	PSP	Regulatory	NCU08779	HO	VA
rgb1	120545	PSP	Regulatory	NCU09377	HE	VA
rga1	77135	PSP	Regulatory	NCU03786	HE	IV
tap42a	74861	PSP	Regulatory	NCU08268	HE	IV
sit4a	35316	PSP	Regulatory	NCU15835	HO	VA
cnb1	52130	PSP	Regulatory	NCU03833	HE	IV
csp6	122050	Asp-	NIF like	NCU08380	HO	IV
tim50	28199	Asp-	NIF like	NCU02943	HE	IV
ptpa2	120498	PTPA	PTPA	NCU03269	HO	VA
ptpa1	79850	PTPA	PTPA	NCU04810	HO	IV
ppi1	119724	PP	PPI	NCU01670	HE	IV

KO: knockout; HO: homokaryon; HE: heterokaryon; NA: not available; VA: viable; IV: inviable

Table S2. Copy number analysis of deletion cassettes in mutant strains

Strains	Mutant code	Number of deletion cassettes integrated
**Δ*pp2a*/TR_56872**	3a2a	1
	4a2a	1
**Δ*rgb1*/TR_120545**	24a2b	1
	24b2a	1
**Δ*sit4a*/TR_35316**	2b2a	1
**Δ*rgc1*/TR_123502**	3a2a	1
	9a2a	4
**Δ*pzl1*/TR_79535**	5a2a	1
	10a2a	1
**Δ*pph5*/TR_21256**	5b2a	1
**Δ*pph8*/TR_81164**	10a2a	1
	10b2a	2
**Δ*pph5-2*/TR_124001**	87a2a	1
	92a2a	1
**Δ*pph9*/TR_58587**	1a2a	2
	3a2a	2
	7a2a	1
**Δ*pty7*/TR_111754**	#6	1
	#7	1
**Δ*pty8*/TR_65499**	6a2a	1
	10a2a	1
**Δ*dsp4*/TR_64938**	26a2a	1
	31b2b	2
**Δ*dsp1*/TR_80292**	9b2a	1
**Δ*pty5*/TR_53525**	19a2a	1
**Δ*pty4*/TR_23417**	2a2a	1
	6a2a	1

strains	osmotic stress				oxidative stress	
	sorbitol		NaCl		menadione	
	LL	DD	LL	DD	LL	DD
**WT**	1,00	1,00	1,00	1,00	1,00	1,00
**Δ*pp2a* /TR_56872**	▽ 0,65	▽ 0,89	▽ 0,51	▽ 0,28	▽ 0,29	▽ 0,22
**Δ*rgb1* /TR_120545**	0,94	▽ 0,72	0,90	▽ 0,66	▽ 0,78	▽ 0,85
**Δ*sit4a* /TR_35316**	▽ 0,49	▽ 0,53	▽ 0,44	▽ 0,64	▽ 0,26	▽ 0,43
**Δ*rgc1* /TR_123502**	▽ 0,72	▽ 0,77	0,96	▽ 0,76	▽ 0,63	▽ 0,73
**Δ*pzl1* /TR_79535**	▽ 0,78	▽ 0,64	0,91	▽ 0,76	△ 1,10	▽ 0,86
**Δ*pph5* /TR_21256**	0,91	▽ 0,74	▽ 0,50	▽ 0,51	▽ 0,92	▽ 0,39
**Δ*pph8* /TR_81164**	▽ 0,57	▽ 0,70	▽ 0,55	▽ 0,59	▽ 0,39	▽ 0,50
**Δ*pph5-2* /TR_124001**	▽ 0,80	▽ 0,83	▽ 0,76	▽ 0,73	▽ 0,65	▽ 0,88
**Δ*pph9* /TR_58587**	▽ 0,70	▽ 0,66	▽ 0,82	▽ 0,67	▽ 0,57	▽ 0,89
**Δ*pty7* /TR_111754**	▽ 0,88	▽ 0,90	0,91	▽ 0,66	▽ 0,68	1,01
**Δ*pty8* /TR_65499**	0,90	▽ 0,80	0,96	▽ 0,83	△ 1,11	▽ 0,85
**Δ*dsp4* /TR_64938**	1,05	0,86	▽ 0,74	▽ 0,71	▽ 0,88	▽ 0,47
**Δ*dsp1* /TR_80292**	▽ 0,80	▽ 0,87	▽ 0,79	0,90	0,95	0,82
**Δ*pty5* /TR_53525**	0,90	▽ 0,84	▽ 0,85	▽ 0,83	▽ 0,57	0,99
**Δ*pty4* /TR_23417**	1,03	0,96	0,98	▽ 0,86	0,94	▽ 0,84

Figure S1. Hyphal extension of protein phosphatase deletion strains in the presence of chemicals imposing osmotic or oxidative stress. Values are normalized to wild-type. Statistically significant results for deletion strains compared to wild-type are marked with a triangle (p-value <0.05) indicating up- (green) or down-regulation (red). Values represent means of at least three biological replicates.
