# Supplementary figures and images for: Protein phosphatases regulate growth, development, cellulases and secondary metabolism in Trichoderma reesei
Source: Sci Rep. 2019 Jul 29;9:10995. doi: 10.1038/s41598-019-47421-z (PMC6662751; doi:10.1038/s41598-019-47421-z)

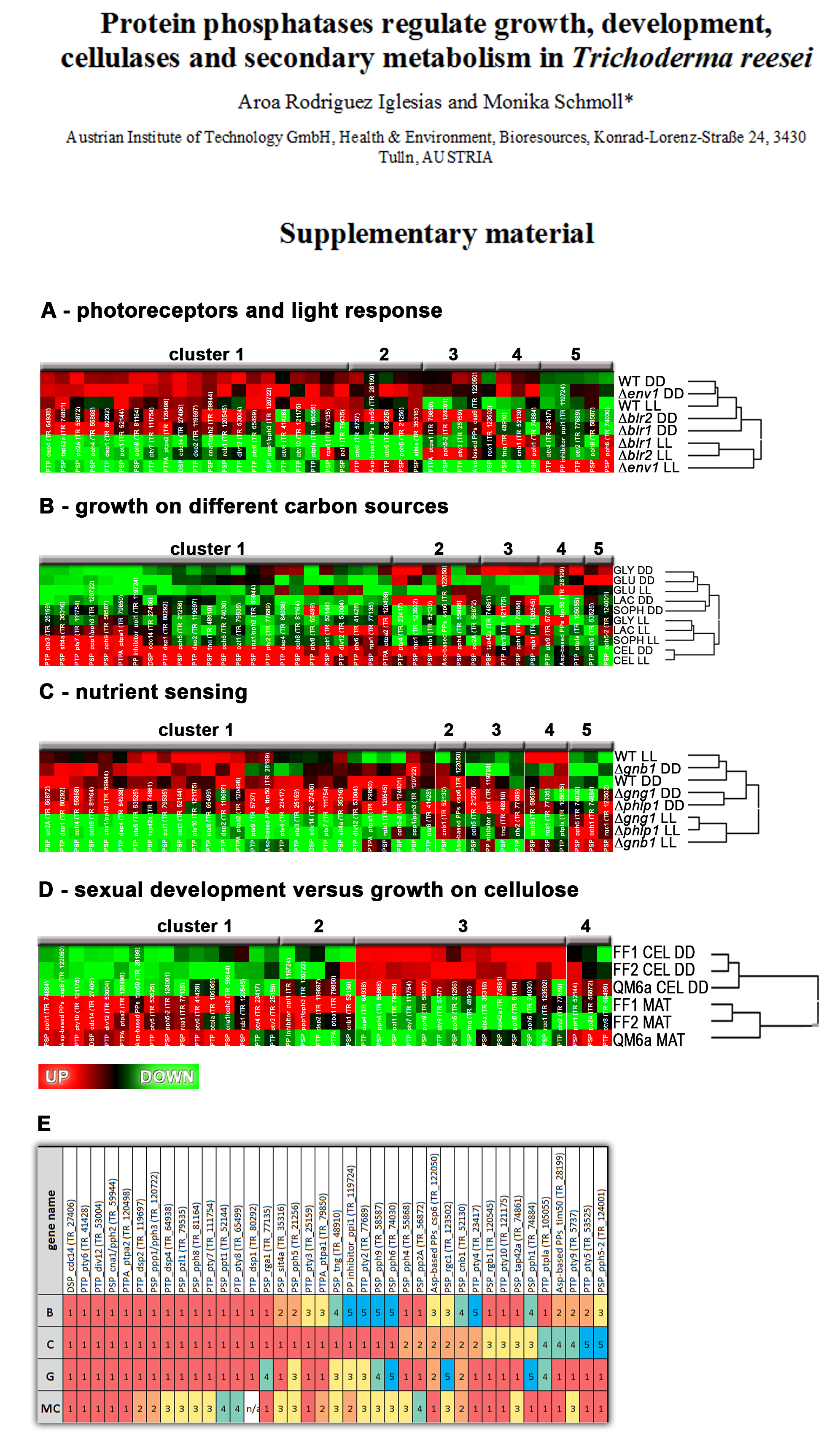

Supplement: Supplementary file 3 — Additional file 3 [file 41598_2019_47421_MOESM3_ESM.tif]
